# Gastric stump carcinoma as a long-term complication of pancreaticoduodenectomy: report of two cases and review of the English literature

**DOI:** 10.1186/s12876-017-0682-x

**Published:** 2017-11-22

**Authors:** Morgane Bouquot, Safi Dokmak, Louise Barbier, Jérôme Cros, Philippe Levy, Alain Sauvanet

**Affiliations:** 1 Department of Hepatic and Pancreatic Surgery, Pôle des Maladies de l’Appareil Digestif, Hospital Beaujon, AP-HP, University Paris Diderot, 100 Boulevard du Maréchal Leclerc, 92110 Clichy, France; 2 Department of Pathology, Hospital Beaujon, AP-HP, University Paris Diderot, 92110 Clichy, France; 3 Department of Gastroenterology and Pancreatology, Pôle des Maladies de l’Appareil Digestif, Hospital Beaujon, AP-HP, University Paris Diderot, 92110 Clichy, France

**Keywords:** Gastric stump carcinoma, Signet-ring cell carcinoma, Pancreaticoduodenectomy

## Abstract

**Background:**

Gastric stump carcinoma is an exceptional and poorly known long-term complication after pancreaticoduodenectomy.

**Cases presentation:**

Two patients developed gastric stump carcinoma 19 and 10 years after pancreaticoduodenectomy for malignant ampulloma and total pancreaticoduodenectomy for pancreatic adenocarcinoma, respectively. Both patients had pT4 signet-ring cell carcinoma involving the gastrojejunostomy site that was revealed by bleeding or obstruction. Patient 1 is alive and remains disease-free 36 months after completion gastrectomy. Patient 2 presented with peritoneal carcinomatosis and died after palliative surgery. We identified only 3 others cases in the English literature.

**Conclusions:**

Prolonged biliary reflux might be the most important risk factor of gastric stump carcinoma following pancreaticoduodenectomy. Its incidence might increase in the future due to prolonged survival observed after pancreaticoduodenectomy for benign and premalignant lesions.

## Background

Several major changes have occurred in the past decade in patients undergoing PD. A better perioperative management allowed a decrease in postoperative mortality below 5% in high-volume centers [1]. Long-term survival after PD for pancreatic ductal adenocarcinoma (PDAC) and periampullary malignancies has improved due to better preoperative selection and adjuvant treatment [2]. Lastly, PD has been more and more frequently performed for benign and potentially malignant disease, including Intraductal Papillary Mucinous Neoplasms [1]. Consequently, both surgeons and gastroenterologists must be aware of long-term results of PD including pancreatic insufficiency and gastrointestinal disorders. Although PD usually includes distal gastrectomy with gastrojejunostomy, long-term risk of gastric stump carcinoma (GSC) after PD is much less known than after partial gastrectomy. Indeed, incidence of GSC after isolated partial gastrectomy is estimated to 1–2% [3, 4] whereas GSC after PD has been exceptionally reported [5–7]. We herein described two new patients who developed GSC 10 and 19 years following PD.

## Cases presentation

### Case 1

A 57-year old male patient presented with upper gastrointestinal (GI) bleeding. He underwent PD with antrectomy for ampullary adenocarcinoma 19 years before. Reconstruction consisted in pancreaticogastrostomy, hepaticojejunostomy and gastrojejunostomy. The gastrojejunostomy was performed downstream on the jejunum. Post-operative course was uneventful. Pathologic examination revealed a pT2N0MxR0 ampullary adenocarcinoma. *Helicobacter Pylori* (HP) infection was present but diagnosed retrospectively at the time of upper GI bleeding. The patient received adjuvant chemotherapy and follow-up with CT-scan and tumor markers was discontinued 5 years after PD.

**Fig. 1:**
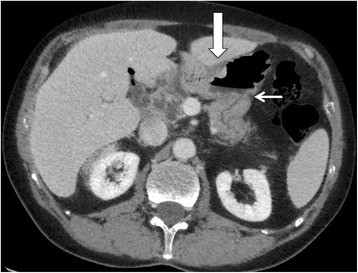
CT scan with contrast injection: axial view. The gastric wall is thickened (large arrow) in front of the pancreaticogastrostomy (thin arrow)

**Fig. 2:**
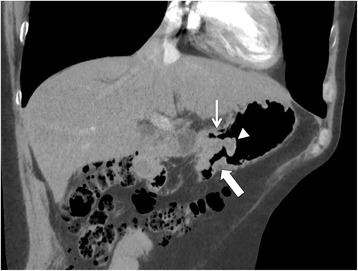
CT scan with contrast injection: coronal view. Both afferent (thin arrow) and efferent (large arrow) jejunal loops are visible close to the gastrojejunostomy site, where the gastric cancer (arrowhead) protrudes into the gastric lumen

At the time of presentation with upper GI bleeding, endoscopy revealed an ulcer on the gastric side of gastrojejunostomy, which was treated by local hemostasis and intravenous proton pump inhibitors (PPI). Pancreaticogastrostomy site was normal. Endoscopy with biopsies performed 3 weeks later revealed signet-ring cell adenocarcinoma. At endoscopic ultrasound, tumor was classified as uT1-uT2N0, and the pancreatic remnant was normal. CT-scan revealed gastric wall thickening close to gastrojejunostomy (Figs. 1 and 2) with no distant metastases. Upfront resection was decided due to presentation with bleeding and the presumed limited tumor stage. Completion total gastrectomy was performed with removal of all anastomoses and reconstruction by hepaticojejunostomy, pancreaticojejunostomy and oesophagojejunostomy on two separate loops. Postoperative course was uneventful. Pathologic examination revealed a T4N0MxR0 signet-ring cell carcinoma with peri-neural invasion but no vascular emboli. In the gastric mucosa, foci of high-grade dysplasia were present but no HP infection. The patient received adjuvant chemotherapy with 5 fluorouracil, folinic acid and oxaliplatin followed by chemo-radiation therapy. The patient remained disease-free 36 months after resection with stable weight under oral enzymes and dietary supplement.

### Case 2

A 78-year old female presented with upper GI obstruction. Ten years before she underwent total PD with antrectomy for PDAC. Total pancreatectomy was justified by presence of invasive tumor on successive intraoperative frozen sections of the transection margins. Reconstruction included hepaticojejunostomy and gastrojejunostomy downstream on the first jejunal loop. Post-operative course was uneventful. Pathologic examination revealed a pT2N1(2+/15) MxR0 PDAC with peri-neural invasion but neither vascular invasion nor HP infection. The patient received adjuvant chemotherapy with gemcitabine. Since surgery, she received pancreatic enzymes, insulin and PPI, and had regular follow-up. Nine years after total pancreatectomy, the patient presented with anemia and a gastrojejunostomy ulcer was diagnosed at endoscopy. The ulcer was attributed to inadvertent PPI discontinuation, while biopsies revealed inflammatory gastric mucosa with no dysplasia.

**Fig. 3:**
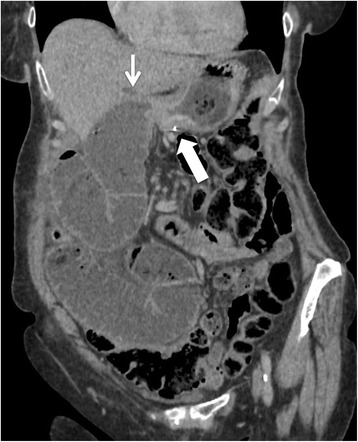
CT without contrast injection: frontal reconstruction showing gastric wall thickening at the gastrojejunostomy site (large arrow). The afferent jejunal loop is dilated with upstream biliary dilatation (thin arrow)

One year after being diagnosed with an ulcer, the patient presented with vomiting and weight loss. CT scan (Fig. 3) showed dilatation of the afferent jejunal loop with thickening of the gastric wall suggestive of gastrojejunostomy stenosis and no signs of PDAC recurrence. Endoscopically, GJ was stenosed and ulcerated. Biopsies revealed a signet-ring cell adenocarcinoma. Endoscopic ultrasound and 18-FDG-PET scan showed tumor limited to the GJ without lymph nodes or distant metastases. Peritoneal carcinomatosis was diagnosed intraoperatively and confirmed by frozen sectioning; hence, only palliative partial gastrectomy with redo-gastrojejunostomy were performed. The patient died at POD 9 from peritonitis due to gastrojejunostomy leakage. Pathologic examination confirmed a pT4N2M1R1 signet-cell carcinoma with peritoneal carcinomatosis, without HP infection.

## Discussion and conclusions

GSC is defined as a gastric carcinoma occurring more than 5 years after distal gastrectomy for benign disease [3, 4, 8]. More rarely, GSC occurs very late after partial gastrectomy for distal gastric carcinoma [3, 9]. The two cases of GSC following PD we reported herein were similar to GSC following distal gastrectomy because: a) PD included distal gastrectomy and reconstruction with hepaticojejunostomy and gastrojejunostomy on the same jejunal loop, that is similar to the Billroth II reconstruction which is supposed to increase the risk of GSC after distal gastrectomy [3, 8]; b) delay of diagnosis of GSC was very long (19 and 10 years after PD respectively), far from the usual delay of ampulloma or PDAC recurrence after PD suggesting that GSC was actually a second cancer; c) both GSC involved the gastrojejunostomy site, a frequent finding in GSC following distal gastrectomy [8]; d) pathologically, both GSC were signet-ring cell carcinoma and clearly differed from ampullary and pancreatic carcinomas. We identified in the English literature three other previously published cases of GSC occurring 4, 4 and 5 years for cholangiocarcinoma in two patients and PDAC in one, respectively [5–7] (table 1). Furthermore, Mihara et al. reported 5 other cases from the Japanese literature occurring 2 to 6 years after pylorus-preserving PD for various indications, including benign and malignant diseases [7]. Considering these arguments, PD should be actually considered at risk for long-term occurrence of GSC.

**Table 1:** Reported cases of gastric stump carcinoma following pancreaticoduodenectomy (English literature)

Author	Indication for PD	Resection type	Delay until GSC (years)	Pathology of GSC	Survival after surgery for GSC
Manabe 2001 [5]	Distal cholangioCa.	PD	4,5	AdenoCa. + lymphoma	Dead (7 mo.)
Mihara 2005 [7]	Distal cholangioCa.	PPPD	4	Moderately differentiated Ca.	Dead (10 mo.)
Kassahun 2008 [6]	PDAC	PD	5	Signet-ring cell Ca.	Alive (4 years)
Case 1	Ampullary adenoCa.	PD	19	Signet-ring cell Ca.	Alive (36 mo.)
Case 2	PDAC	TP	10	Signet-ring cell Ca.	Dead (POD 9)

*PD* pancreaticoduodenectomy, *GSC* Gastric stump carcinoma, *Ca.* carcinoma, *PDAC* pancreatic ductal adenocarcinoma, *PPPD* pylorus-preserving PD, *TP* total pancreatectomy, *POD* post-operative days

Pathophysiology of GSC is partially known. The predominant mechanism following GSC following distal gastrectomy could be a prolonged bilio-pancreatic reflux as suggested by the higher prevalence of Billroth II reconstruction in patients with GSC and its predominant para-anastomotic localization [3, 4, 8, 9]. Mihara et al. suggested that pancreatic reflux due to pancreaticogastrostomy could also favor GSC after PD [7]. Others suggested mechanisms are gastric mucosa infection by HP [10] or Epstein Barr Virus [11], mucosa molecular abnormalities [9] or more rarely genetic predisposition as documented in patients with familial adenomatous polyposis [12]. The two cases we reported herein suggest that biliary reflux is sufficient to promote GSC since the second patient developed GSC 10 years after total PD, without pancreatic reflux and HP infection. The later was present only on the PD specimen of the first patient.

Prevention of GSC following PD is hypothetical. Reconstruction using a separate Roux-en-Y loop has been proposed to avoid bilio-gastric reflux, but could increase the risk of GJ ulceration since alkaline pancreatic and biliary flows diverted from the acid gastric lumen, as reported with Roux-en-Y loop after distal gastrectomy for cancer [13]. Also, it is still unproven that Roux-en-Y reconstruction decreases the risk of GSC comparatively to Billroth I or II reconstruction. Proton pump inhibitors could prevent marginal ulcer but its benefit on the GSC risk is speculative. HP infection should be screened and HP should be eradicated whenever present. Pylorus preservation could theoretically limit biliary reflux into gastric lumen and therefore the GSC risk. However, one retrospective study did not demonstrate a lower incidence of bile gastritis after PD with pylorus preservation comparatively to the Whipple procedure [14]. More importantly, the 5 Japanese cases reported by Mihara et al. [7] had PD with pylorus-preservation, suggesting that this technical variant does not prevent the GSC risk.

Curative treatment of GSC following PD is challenging. Neoadjuvant chemotherapy is theoretically indicated but could be ineffective in case of poorly differentiated carcinoma [15] which was present in our two patients and in one previously reported case [6], and also represent half of cases of GSC following distal gastrectomy [9]. Due to the lack of screening, GSC following PD is frequently revealed by a complication. Technically, completion gastrectomy with lymph node dissection is necessary, and should be combined with proximal jejunum resection when lymph node metastases involve the mesentery [8]. Nutritional support is important, particularly if adjuvant treatment is performed.

In conclusion, GSC is a rare complication occurring several years after PD. GSC is frequently poorly differentiated and located at the gastrojejunostomy site. Its treatment is challenging, Mostly, its incidence may increase in the future due to prolonged survival observed after PD for benign and premalignant lesions. We suggest that long-term follw-up of patients undergoing PD should be more precisely evaluated. Databases from very high-volume centers or even nationwide databases could be helpful for this purpose. Also, upper GI symptoms in patients who have a several years follow-up after PD should lead to raise the diagnosis of GSC.

## Abbreviations

18-FDG-PET scan: Positron-emission tomography coupled with CT after 18-fluorodeoxyglucose (18-FDG) injection
GSC: Gastric stump carcinoma
HP: *Helicobacter Pylori*
PD: Pancreaticoduodenectomy
PDAC: Pancreatic ductal adenocarcinoma
